# The incidence and mortality of major cancers in China, 2012

**DOI:** 10.1186/s40880-016-0137-8

**Published:** 2016-08-02

**Authors:** Wanqing Chen, Rongshou Zheng, Hongmei Zeng, Siwei Zhang

**Affiliations:** National Office for Cancer Prevention and Control & National Central Cancer Registry, National Cancer Center/Cancer Hospital, Chinese Academy of Medical Sciences and Peking Union Medical College, Beijing, 100021 P. R. China

**Keywords:** Incidence, Mortality, Cancer registry, Epidemiology, China

## Abstract

**Background:**

The National Central Cancer Registry (NCCR) collected population-based cancer registration data in 2012 from local registries and estimated the cancer incidence and mortality in China.

**Methods:**

In the middle of 2015, 261 cancer registries submitted reports on new cancer cases and deaths occurred in 2012. Qualified data from 193 registries were used for analysis after evaluation. Crude rates, number of cases, and age-standardized rates stratified by area (urban/rural), sex, age group, and cancer type were calculated according to the national population in 2012.

**Results:**

The covered population were 198,060,406 from 193 qualified cancer registries (74 urban and 119 rural registries). The major indicators of quality control, percentage of cases morphologically verified (MV%), death certificate-only cases (DCO%), and the mortality to incidence (M/I) ratio, were 69.13%, 2.38%, and 0.62, respectively. It was estimated that there were 3,586,200 new cancer cases and 2,186,600 cancer deaths in 2012 in China with an incidence of 264.85/100,000 [age-standardized rate of incidence by the Chinese standard population (ASRIC) of 191.89/100,000] and a mortality of 161.49/100,000 [age-standardized rate of mortality by the Chinese standard population (ASRMC) of 112.34/100,000]. The ten most common cancer sites were the lung, stomach, liver, colorectum, esophagus, female breast, thyroid, cervix, brain, and pancreas, accounting for approximately 77.4% of all new cancer cases. The ten leading causes of cancer death were lung cancer, liver cancer, gastric cancer, esophageal cancer, colorectal cancer, pancreatic cancer, female breast cancer, brain tumor, leukemia, and lymphoma, accounting for 84.5% of all cancer deaths.

**Conclusions:**

Continuous cancer registry data provides basic information in cancer control programs. The cancer burden in China is gradually increasing, both in urban and rural areas, in males and females. Efficient cancer prevention and control, such as health education, tobacco control, and cancer screening, should be paid attention by the health sector and the whole society of China.

The National Central Cancer Registry (NCCR) called for data in 2012 from all population-based cancer registries in China, and the datasets were used to update the annual estimation for new cancer cases and deaths in China after data evaluation and analysis.

There were 261 cancer registries, located in 32 provinces, autonomous regions, or municipalities, submitted cancer data in 2012. All data were checked and evaluated according to the criteria of data quality from NCCR, and 193 registries’ data were qualified and accepted for this updated cancer statistics in China in 2012. Cancer registries collected information on new cancer cases from hospitals, community health centers, medical insurance agents, and the Vital Statistics System (for cases only identified by death certification). Registries obtained information on cancer deaths from the death surveillance system, which collects death information from hospitals and the Civil Administration Bureau with available cremation records. Population information was collected from local statistical bureau or household register department in local public security bureau. Proportion of morphological verification (MV%), percentage of cancer cases identified with death certification only (DCO%), mortality to incidence (M/I) ratio, percentage of uncertified cancer (UB%), and percentage of cancer with undefined or unknown primary site (secondary) (O&U%) were used to evaluate the completeness, validity, and reliability of cancer statistics. Pooled data were stratified by area (urban/rural), sex, age group, and cancer site. Cancer incident cases and deaths were estimated using age-specific rates, which were stratified by area (urban/rural), sex (male/female), and age (grouped by 0, 1–4, 5–9,…, 80–84, 85-year old and above). The Chinese population census in 2000 and the Segi’s population were used for age-standardized rates (ASR) of incidence/mortality. The national incidences and mortalities were estimated using stratified rates and modelled national population in 2012.

All 193 cancer registries (74 urban and 119 rural registries) covered a total of 198,060,406 population (100,450,109 in urban areas and 97,610,297 in rural areas). The overall indicators MV%, DCO%, and M/I ratio were 69.13%, 2.38%, and 0.62, respectively. They were 70.63%, 2.63%, and 0.59 in urban registries, compared to 67.31%, 2.09%, and 0.65 in rural registries. The estimates of new cases and deaths were 3,586,200 and 2,186,600 in 2012, respectively.

The crude cancer incidence in China was 264.85/100,000 (289.30/100,000 in males and 239.15/100,000 in females), and age-standardized rates of incidence by the Chinese standard population (ASRIC) and by the world standard population (ASRIW) were 191.89/100,000 and 187.83/100,000 with a cumulative rate (for the whole population of 0–74 years old) of 21.82%. The crude incidence and ASRIC were 277.17/100,000 and 195.56/100,000 in urban areas and were 251.20/100,000 and 187.10/100,000 in rural areas. Cancer incidence was relatively low before 40 years old, after then increased dramatically, and finally peaked after 80 years old and slightly decreased after 85 years old. The pattern was similar between urban and rural areas (Table 1).

**Table 1:** Cancer incidence in China in 2012 (This table has been published previously and is reproduced with permission from the *Chinese Journal of Cancer Research*, see Ref. [3])

Areas	Sex	New cases (×10^3^)	Crude incidence (1/10^5^)	ASRIC (1/10^5^)	ASRIW (1/10^5^)	Cumulative rate (%)^a^
All areas	Both	3586.2	264.85	191.89	187.83	21.82
	Male	2007.6	289.30	216.17	214.33	25.39
	Female	1578.6	239.15	170.08	163.81	18.32
Urban areas	Both	1973.0	277.17	195.56	190.88	21.91
	Male	1062.0	292.31	212.68	210.63	24.71
	Female	911.0	261.39	181.30	174.00	19.28
Rural areas	Both	1613.2	251.20	187.10	183.91	21.67
	Male	945.6	286.00	220.03	218.42	26.14
	Female	667.6	214.28	156.14	151.35	17.14

*ASRIC* age-standardized rate of incidence by the China population in 2000, *ASRIW* age-standardized rate of incidence by the Segi’s population

^a^ The cumulative rate of the whole population of 0–74 years old

The crude cancer mortality in China was 161.49/100,000 (198.99/100,000 in males and 122.06/100,000 in females), age-standardized rates of mortality by the Chinese standard population (ASRMC) and by the world standard population (ASRMW) were 112.34/100,000 and 111.25/100,000, and the cumulative mortality (for the whole population of 0–74 years old) was 12.61%. The crude mortality and ASRMC were 159.00/100,000 and 107.23/100,000 in urban areas and were 164.24/100,000 and 118.22/100,000 in rural areas. The cancer mortality was relatively low before 45 years old and then dramatically increased, reaching the peak after 85 years old. The mortality in rural areas was highest in the age group of 80–84 years old. The age-specific mortality in males was lower in urban areas than in rural areas in most of age groups before 80 years old (Table 2).

**Table 2:** Cancer mortality in China in 2012 (This table has been published previously and is reproduced with permission from the *Chinese Journal of Cancer Research*, see Ref. [3])

Areas	Sex	Deaths (×10^3^)	Crude mortality (1/10^5^)	ASRMC (1/10^5^)	ASRMW (1/10^5^)	Cumulative rate (%)^a^
All areas	Both	2186.6	161.49	112.34	111.25	12.61
	Male	1380.9	198.99	146.51	145.57	16.57
	Female	805.7	122.06	80.00	78.89	8.66
Urban areas	Both	1131.8	159.00	107.23	106.13	11.70
	Male	706.2	194.37	138.60	137.74	15.30
	Female	425.7	122.14	77.74	76.55	8.18
Rural areas	Both	1054.8	164.24	118.22	117.06	13.63
	Male	674.7	204.06	155.46	154.28	17.99
	Female	380.1	121.98	82.64	81.60	9.21

*ASRMC* age-standardized rate of mortality by the China population in 2000, *ASRMW* age-standardized rate of mortality by the Segi’s population

^a^ The cumulative rate of the whole population of 0–74 years old

Lung cancer was the most common cancer in all areas, followed by gastric, liver, colorectal, and esophageal cancers with estimated new cases of 704,800, 423,500, 366,100, 331,300, and 286,700, respectively. Lung cancer was the most frequently diagnosed cancers in males, followed by gastric, liver, esophageal, and colorectal cancers; breast cancer was the most common cancer in females, followed by lung, colorectal, gastric, and cervical cancers (Table 3; Figs. 1, 2). The ten most common cancers accounted for approximately 77.4% of all cancer cases in urban and rural areas. In urban areas, lung cancer was the most frequently diagnosed cancer, followed by colorectal, gastric, female breast, and liver cancers. Lung cancer had been becoming the most frequently diagnosed cancer (326,600 new cases with an incidence of 50.86/100,000), followed by gastric, esophageal, liver, and colorectal cancers.

**Table 3:** Top ten cancer incidences in China in 2012 (This table is modified from a previous publication with permission, see Ref. [3])

*CNS* central nervous system

**Fig. 1:**
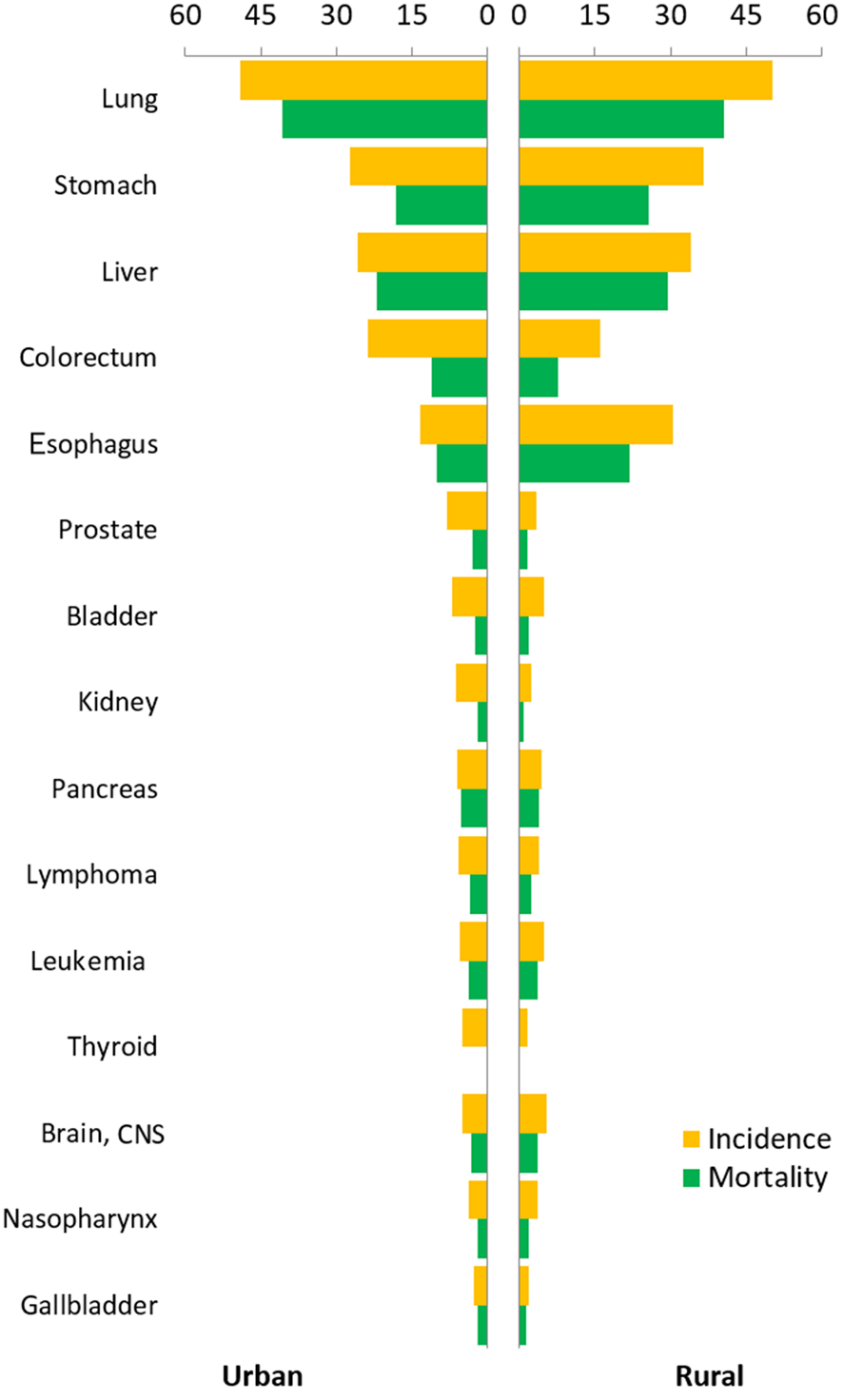
Cancer incidence and mortality in urban and rural areas for males in China in 2012 (1/100,000). *CNS* central nervous system

**Fig. 2:**
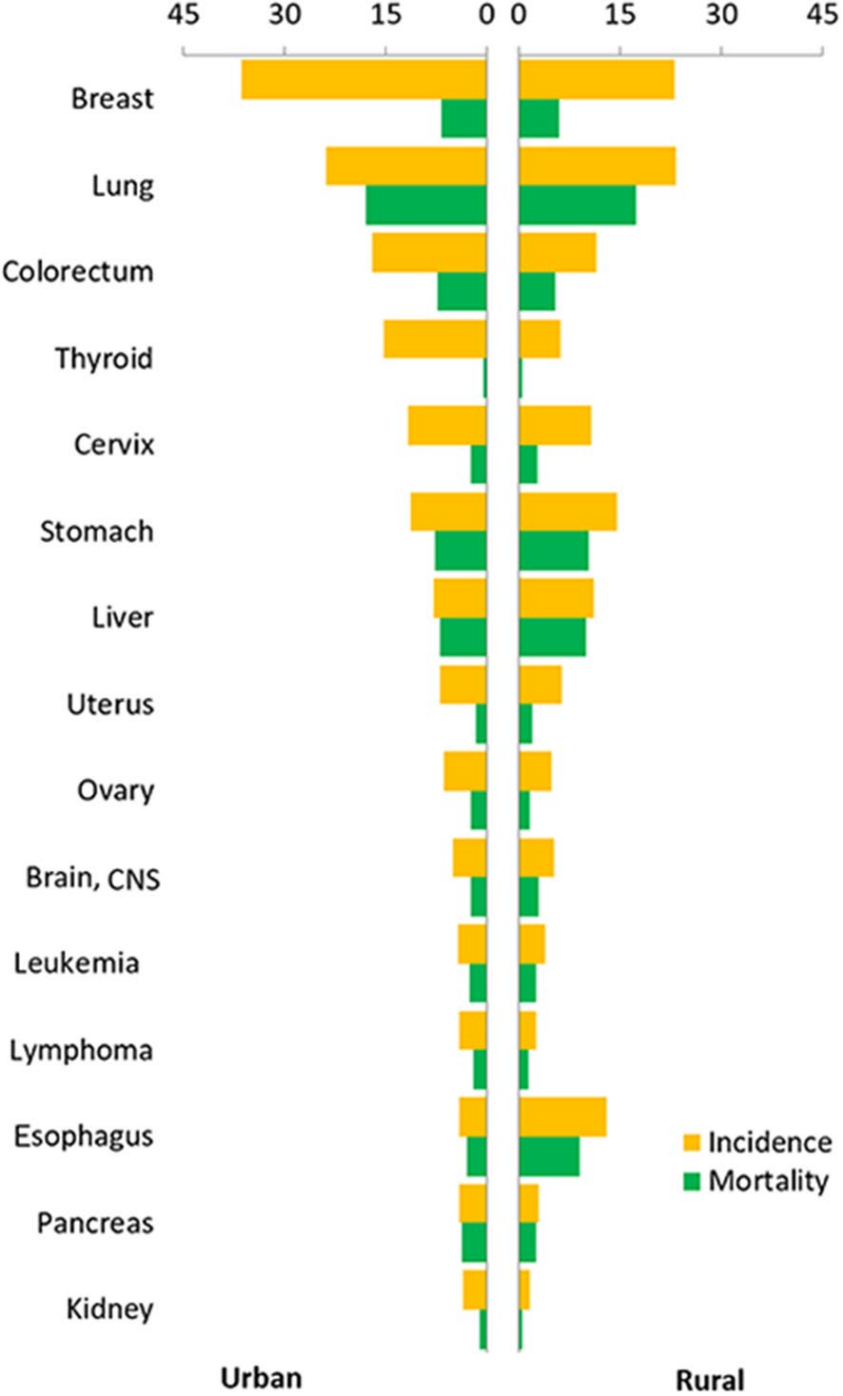
Cancer incidence and mortality in urban and rural areas for females in China in 2012 (1/100,000). *CNS* central nervous system

Lung cancer was the first leading cause of cancer death in China, followed by liver, gastric, esophageal, and colorectal cancers with estimated deaths of 569,400, 321,200, 298,500, 210,900, and 159,300, respectively. In males, lung cancer was the leading cause of cancer death, followed by liver, gastric, esophageal, and colorectal cancers; whereas in females, lung cancer was still the leading cause of cancer death, followed by gastric, liver, colorectal, and esophageal cancers (Table 4; Figs. 1, 2). The top ten leading causes of cancer death accounted for approximately 84.5% of all cancer deaths. The cancer patterns showed difference between urban and rural areas and between males and females. The leading causes of cancer death in rural areas were lung, liver, gastric, esophageal, and colorectal cancers, whereas the leading causes of cancer death in urban areas was lung, liver, gastric, colorectal, and esophageal cancers.

**Table 4:** Top ten cancer mortalities in China in 2012 (This table is modified from a previous publication with permission, see Ref. [3])

*CNS* central nervous system

Compared with the figures in 2011 [1] and 2010 [2], cancer burden kept increasing, mostly because of the population increase and aging. Timely report of cancer registry provides basic information for policymakers, researchers, and clinicians. With the registry data getting more accurate, it plays more important role in cancer control in China.
